# Physiological and Dual Transcriptional Analysis of Microalga *Graesiella emersonii–Amoeboaphelidium protococcarum* Pathosystem Uncovers Conserved Defense Response and Robust Pathogenicity

**DOI:** 10.3390/ijms222312847

**Published:** 2021-11-27

**Authors:** Yi Ding, Zhongjie Wang, Yali Wang, Yahong Geng, Xiaobin Wen, Yeguang Li

**Affiliations:** 1 CAS Key Laboratory of Plant Germplasm Enhancement and Specialty Agriculture, Wuhan Botanical Garden, Chinese Academy of Sciences, Wuhan 430074, China; dingyi@wbgcas.cn (Y.D.); wzjihb@gamil.com (Z.W.); wangyali20@mails.ucas.ac.cn (Y.W.); yahong@wbgcas.cn (Y.G.); 2 Center of Economic Botany, Core Botanical Gardens, Chinese Academy of Sciences, Wuhan 430074, China; 3 University of Chinese Academy of Sciences, Beijing 100049, China

**Keywords:** oleaginous microalgae, *Amoeboaphelidium protococcarum*, dual RNA-seq, host defense response, pathogenicity

## Abstract

The underlying mechanisms of microalgal host–pathogen interactions remain largely unknown. In this study, we applied physiological and simultaneous dual transcriptomic analysis to characterize the microalga *Graesiella emersonii–Amoeboaphelidium protococcarum* interaction. Three infection stages were determined according to infection rate and physiological features. Dual RNA-seq results showed that the genes expression of *G. emersonii* and *A. protococcarum* were strongly dynamically regulated during the infection. For microalgal hosts, similar to plant defense response, the expression of defense genes involved in the pattern recognition receptors, large heat shock proteins, and reactive oxygen scavenging enzymes (glutathione, ferritin, and catalase) were significantly upregulated during infection. However, some genes encoding resistance proteins (R proteins) with a leucine-rich repeat domain exhibited no significant changes during infection. For endoparasite *A. protococcarum*, genes for carbohydrate-active enzymes, pathogen–host interactions, and putative effectors were significantly upregulated during infection. Furthermore, the genes in cluster II were significantly enriched in pathways associated with the modulation of vacuole transport, including endocytosis, phagosome, ubiquitin-mediated proteolysis, and SNARE interactions in vesicular transport pathways. These results suggest that *G. emersonii* has a conserved defense system against pathogen and that endoparasite *A. protococcarum* possesses a robust pathogenicity to infect the host. Our study characterizes the first transcriptomic profile of microalgae–endoparasite interaction, providing a new promising basis for complete understanding of the algal host defense strategies and parasite pathogenicity.

## 1. Introduction

Oleaginous microalgae have been suggested as a promising feedstock for biodiesel production [1]. In recent years, oleaginous microalgae cultivation, combined with wastewater treatment and CO_2_ mitigation, has been suggested as an environmentally sustainable process with the production of high value-added bioproducts [2,3]. However, the mass cultivation of microalgae is susceptible to microbial contamination (especially parasitic fungi), which can lead to algal culture crashes [4]. In natural ecosystems, algal parasites are a key driving factor in phytoplankton seasonal successions [5]. In the mass cultivation of microalgae, these parasites can cause microalgal population crashes and trigger further damage to their valuable products [6]. Knowledge about the isolation, identification, and progression of algal parasites is currently growing [7]. However, the underlying mechanisms of algal infection by fungal parasites remain largely unknown due to the lack of stable pathosystems for laboratory investigations [8,9]. 

The oleaginous microalga *Graesiella emersonii* is an industrial strain for lipid production [10], but it is frequently infected by endoparasite *Amoeboaphelidium protococcarum* belonging to the class Aphelidea. The infection progress starts with an amoeboid zoospore attached to an algal cell, followed by the formation of a cyst with a penetration tube [11]. Then, the propagule of *A. protococcarum* is penetrated into the host cell, thus triggering its intracellular development to gradually phagocytize the contents of the algal cell [4]. *A. protococcarum* exhibited high host specificity on *G. emersonii* and *Scenedesmus dimorphus* [7]. In a previous study, we created a stable and laboratory *G. emersonii* and *A. protococcarum* pathosystem. Hence, this pathosystem can enable the investigation of microalgal–fungal parasite interactions. 

In addition, recent studies have characterized the molecular defensive mechanisms of macroalgae using the algal pathosystem. For instance, studies on the brown alga *Ectocarpus siliculosus* and the oomycete *Eurychasma dicksonii* have demonstrated that *E. siliculosus* responds to pathogen infection by strengthening the cell wall and accumulating reactive oxygen species (ROS) and putative halogen metabolism [12]. In 2019, Im et al. identified the genes involved in the interaction of the red alga *Pyropia tenera* and three pathogens using microarray analysis and histochemical methods [13]. Tang et al. identified the genes associated with the defense response of the red alga *Pyropia yezoensis* against the necrotrophic pathogen *Pythium porphyrae* using transcriptomic analysis [14]. However, microalgal responses and defense reactions to pathogen infection at the molecular level have never been investigated.

In this study, physiological and simultaneous dual transcriptomic analyses were conducted to characterize the *G. emersonii*–*A. protococcarum* interaction for the first time. We aimed to understand the host algal defense strategies and algal parasite pathogenicity during the interaction between *G. emersonii* and *A. protococcarum*. These results may provide new insights into microalgal host responses to endoparasites or the identification of pathogen–host interaction genes. 

## 2. Results

### 2.1. Analysis of the Symptom and Features Associated with Infection by A. protococcarum

**Figure 1:**
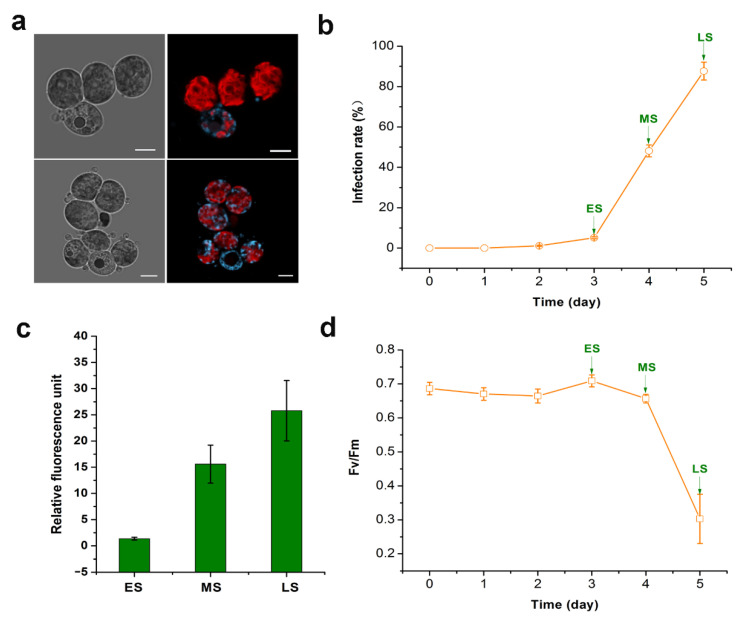
Symptoms and physiological features of *Graesiella emersonii* due to *Amoeboaphelidium protococcarum* infection. (**a**) Typical symptom associated with infection by *A. protococcarum* under fluorescence microscope, scale bars = 5 μm; (**b**) infection rate; (**c**) the relative ROS levels; (**d**) photosynthetic activity (Fv/Fm).

Microscopic observation of the infection progress revealed that *A. protococcarum* was an endoparasite that can occupy the microalgal host cell and replace the host cytoplasm (Figure 1a). In the infection test, the infection rates of *G. emersonii* by *A. protococcarum* gradually increased over time (Figure 1b). In contrast, healthy and uninfected *G. emersonii* cells did not exhibit any symptoms during cultivation. Infection stages were determined based on the infection rate. Briefly, healthy and uninfected *G. emersonii* cells were used as controls (GA). At 3 dpi, the infection rate of *G. emersonii* reached approximately 5% and was defined as early stage infection (ES). At 4 dpi, the infection rate reached approximately 50% and was defined as medium stage (MS). At 5 dpi, the infection rate reached approximately 90% in *G. emersonii* cells and was defined as late stage (LS). Samples of GA, ES, MS, and LS were collected and stored in liquid nitrogen for subsequent dual transcriptomic analysis. 

The ROS content and Fv/Fm values of *G. emersonii* cells during infection were measured, and the results are shown in Figure 1c,d. With the increase in culture time, ROS levels gradually increased. At 1, 2, and 3 dpi, the values of Fv/Fm were not significantly decreased compared with those of the control. At 5 dpi, Fv/Fm values decreased significantly.

### 2.2. A Global View of Transcriptomic Analysis of Microalga and Endoparasite during Their Interaction

**Figure 2:**
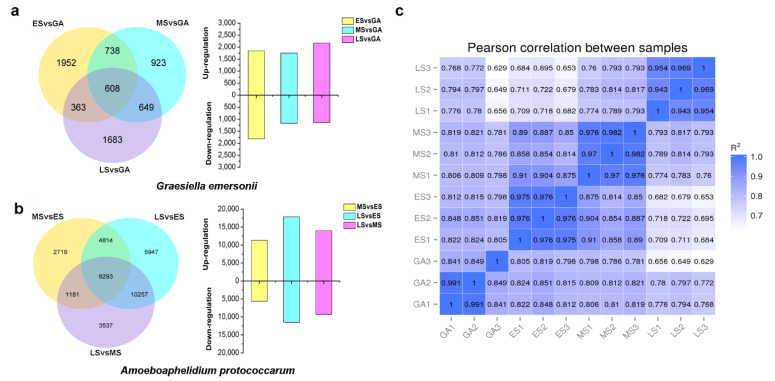
Global view of differentially expressed genes in *G. emersonii* and *A. protococcarum* during infection. (**a**) G. emersonii; (**b**) *A. protococcarum*; (**c**) Pearson correlation coefficients of all 12 samples.

To characterize the gene expression profiles in the host microalga *G. emersonii* and endoparasite *A. protococcarum* during infection, we performed a dual RNA-seq analysis at different infection stages. A total of 102 Gb of clean bases were generated from 12 samples, and sequencing data quality is summarized in Table S1. For the microalgal host, 1843, 1750, and 2164 differentially expressed genes (DEGs) were upregulated, whereas 1818, 1168, and 1139 DEGs were downregulated in ES, MS, and LS compared with GA, respectively (Figure 2a). We found that more genes were upregulated, while fewer genes were downregulated in MS. As the stages of infection progressed, the ratio of upregulated genes to the whole DEGs increased from 50.3% at ES to 65.5% at LS. Figure 2a illustrates that only 608 DEGs overlapped with ES, MS, and LS, suggesting that the microalgal response due to infection is different at the molecular level in three stages. For fungal parasites, a total of 11,352 and 17,819 upregulated DEGs were detected in MS and LS compared with the ES group (Figure 2b). These genes may play a key role in the progression of infection. In addition, Pearson correlation coefficients of the RNA-Seq 12 samples are displayed in Figure 2c. 

### 2.3. Gene Expression Profile of G. emersonii during Infection

**Figure 3:**
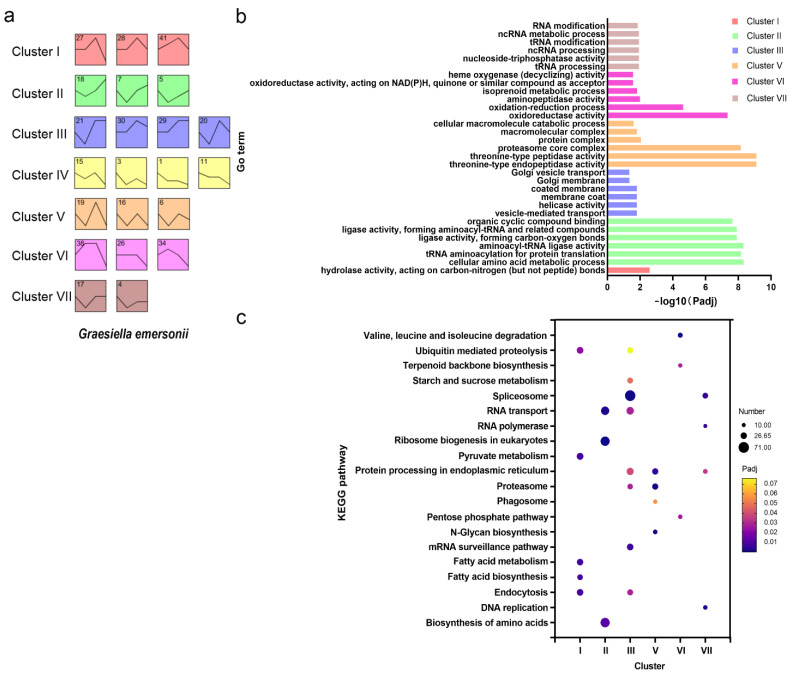
STEM trend and functional enrichment analysis of microalga *G. emersonii* during infection. (**a**) Seven clusters by STEM analysis; (**b**) GO terms enrichment analysis; (**c**) KEGG pathway enrichment analysis.

To further investigate the gene expression profile of *G. emersonii* during infection, the gene expression of the host *G. emersonii* was clustered using the short time-series expression miner (STEM). A total of 23,248 genes were divided into 50 profiles, but only 22 highly significant expression profiles were chosen for further functional analysis (*p* < 0.001) (Figure S1). The 22 profiles were then grouped into seven major clusters based on their expression patterns (Figure 3a). Compared with the GA, cluster I comprised profiles 27, 28, and 41, where the genes were generally upregulated in both ES and MS stages and were not affected in LS stages. Genes in cluster II, combining profiles 18, 7, and 5, were downregulated in the ES stage but upregulated in both the MS and LS stages. Cluster III (profiles 21, 30, 29, and 20) included genes whose expression was generally upregulated but fluctuated. Genes in cluster IV showed an overall downregulation. The genes in cluster V were downregulated but experienced some variability. The majority of genes in cluster VI were upregulated or were stable in the ES and MS stages and downregulated in the LS stage. The genes in cluster VII were generally upregulated in ES and stable in the MS and LS stages.

To elucidate the biological function in each cluster, GO term enrichment and KEGG pathway analyses were conducted for significant enrichment. The top six GO terms for each cluster are shown in Figure 3b. Cluster I, which peaked in expression at the MS stage, was significantly enriched for genes involved in hydrolase activity. The DEGs in cluster II were enriched with many ligase activity-related GO terms, cluster VI was enriched with numerous oxidoreductase-related terms, while cluster IV showed no significant results. 

KEGG pathway analysis showed that seven clusters mapped significantly with 20 pathways (Figure 3c). Notably, cluster I was enriched in the ubiquitin-mediated proteolysis pathway, endocytosis, fatty acid biosynthesis, and fatty acid metabolism. Multiple clusters were enriched with endocytosis, proteasome, protein processing in the endoplasmic reticulum, and spliceosome.

### 2.4. Ubiquitin Mediated Proteolysis and Endocytosis in Response to Infection

**Figure 4:**
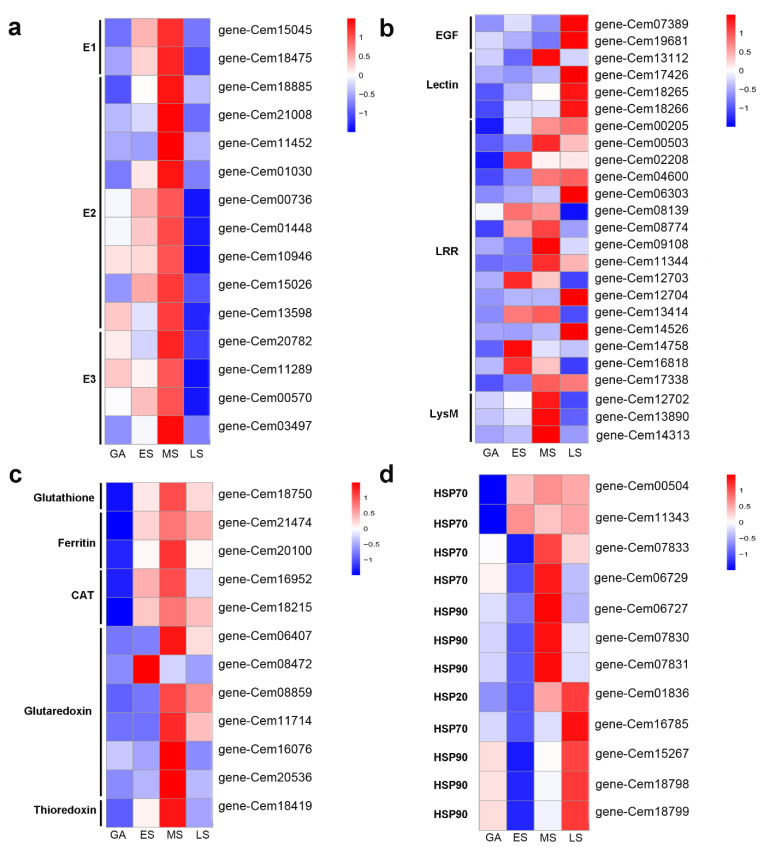
Heatmap of defense-related genes in *G. emersonii* during infection. (**a**) Ubiquitin–proteasome system (UPS)-related genes; (**b**) typical pattern-recognition receptor (PRR)-related genes; (**c)** ROS-related genes; (**d**) heat shock proteins (HSPs)-related genes.

Given the above-mentioned enriched pathways in the microalga *G. emersonii* during infection, we focused on the pathways of ubiquitin-mediated proteolysis and endocytosis. In the transcriptome data, the expression of genes encoding enzyme E1 (gene-Cem18475, gene-Cem15045) was upregulated by 2.2- and 1.1-log_2_ fold changes, respectively (Figure 4a). The expression of genes encoding E2 (gene-Cem18885, gene-Cem21008, gene-Cem11452, and gene-Cem01030) also experienced a significant increase. In contrast, the expression of several genes encoding E3 was downregulated. Moreover, the genes encoding other key enzymes, such as heat shock proteins (HSPs), which are also involved in the endocytosis pathway, were induced. 

### 2.5. Potential Pathogen Receptors and Putative R Proteins in G. emersonii

In *G. emersonii*, genes that contained typical pattern-recognition receptor (PRR) functional domains were searched using Pfam annotation. Epidermal growth factor (EGF)-containing, lectin-containing genes, leucine-rich repeat (LRR)-containing genes, and LysM-containing genes with upregulated expression between one or more infection stages are summarized in Figure 4b. Among these genes, EGF (gene-Cem19681), lectin-containing genes (gene-Cem18265, gene-Cem18266), and LRR-containing genes (gene-Cem02208, gene-Cem04600, gene-Cem11344, gene-Cem14526, gene-Cem14758, gene-Cem16818) were predicted as putative transmembrane proteins using TMHMM v. 2.0. At the same time, LRR-containing genes (gene-Cem08139, gene-Cem08774, gene-Cem12703, gene-Cem13414, and gene-Cem16818) significantly increased in both ES and MS during infection. The LRR-containing gene (gene-Cem02208) was upregulated in ES by 5.6-fold. Moreover, four genes encoding putative R proteins with an LRR domain (gene-Cem07770, gene-Cem16366, gene-Cem22200, and Novel00913) were found, but none were significantly upregulated at the three infection stages. No genes encoding the TIR or NBS domain were detected in the *G. emersonii* transcripts. 

### 2.6. ROS-Related Genes in Response to Infection 

The generation of superfluous ROS due to pathogens may cause oxidative stress and cell damage. *G. emersonii* can protect themselves by various ROS-scavenging enzymes. Our results demonstrate that the expression of genes encoding glutathione (gene-Cem18750), ferritin (gene-Cem21474), and CAT (gene-Cem18215) were upregulated at ES and maintained at high levels in MS and LS (Figure 4c). The genes encoding glutaredoxin (gene-Cem06407, gene-Cem11714) were not significantly modified at ES, but were upregulated in MS and LS. Notably, the gene encoding for ferritin (gene-Cem21474) was upregulated by 7.9 log_2_ fold change at ES, 8.4 log_2_ fold change at MS, and 8.1 log_2_ fold change at LS. The expression of genes encoding glutathione (gene-Cem18750) was also dramatically increased by 5.3 log_2_ fold change at ES, 6.6 log_2_ fold change at MS, and 5.4 log_2_ fold change at MS. These transcriptional results confirmed that the upregulation of genes encoding ROS-scavenging enzymes is one of the defense strategies of *G. emersonii* during infection. 

### 2.7. Some Heat Shock Proteins (HSPs) and Transcription Factors (TFs) Involved during Infection

The results of this study show that 12 genes encoding heat shock proteins were differentially regulated during infection (Figure 4d). Compared with the uninfected control group, five HSP genes (four HSP90 and one HSP70) were upregulated in one or more infection stages. 

RNA-seq analysis revealed 774 differentially expressed TFs in *G. emersonii* (see Table S2). SET, SNF2, GNAT, MYB, AP2-EREBP, and TRAF were the top six differentially expressed TFs. Among them, the identified five TFs that were upregulated during the infection included two SNF2, one PLATZ, one FHA, and one orphan (Table 1).

### 2.8. Gene Expression Profile of A. protococcarum during Infection

**Figure 5:**
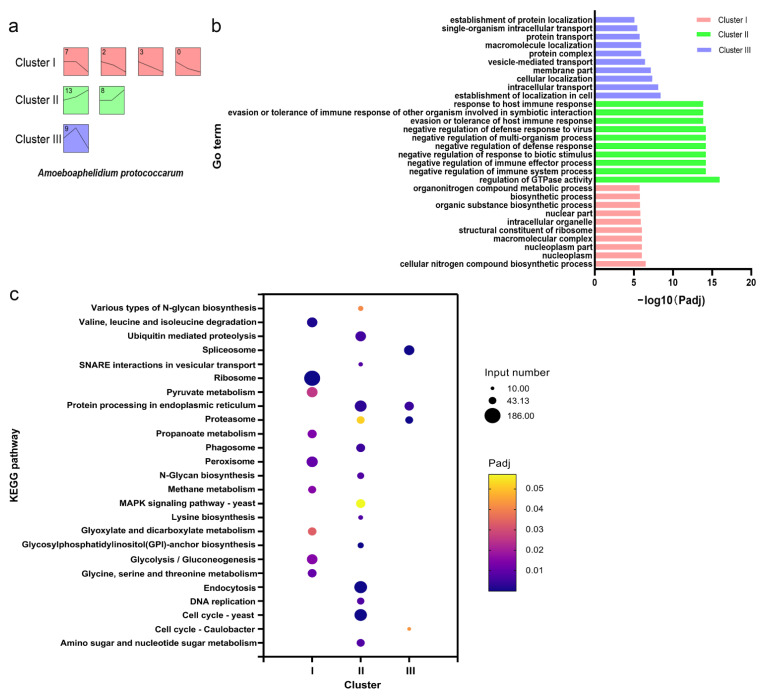
STEM trend and functional enrichment analysis of endoparasite *A. protococcarum* during infection. (**a**) Three clusters by STEM analysis; (**b**) GO terms enrichment analysis; (**c**) KEGG pathway enrichment analysis.

STEM analysis was carried out to elucidate the gene expression profile of *A. protococcarum* during infection. In total, 16 profiles were generated, but only 7 highly significant expression profiles were chosen for further functional analysis (*p* < 0.001) (see Figure S2). The seven profiles were subsequently grouped into three clusters based on their expression patterns (Figure 5a). Cluster I comprised profile 7, 2, 3, and 0, where the genes were generally downregulated compared with those in ES. Genes in cluster II, which combined profiles 13 and 6, were upregulated. Genes in cluster III were upregulated and then downregulated. 

GO term enrichment and KEGG pathway analyses were also conducted for the three clusters of *A. protococcarum*. The top 10 GO terms for each cluster are shown in Figure 5b. The genes involved in macromolecular complex, intracellular organelle, and organic substance biosynthetic processes that tended toward downregulation were related to cluster I. The genes associated with the regulation of GTPase activity and response to host immune that showed an upregulated trend were in cluster II. The genes in cluster III were enriched in the establishment of localization in cells, cellular localization, and vesicle-mediated transport GO terms. These GO terms were both necessary and significant to *A. protococcarum* infection. 

KEGG pathway analysis demonstrated that the three clusters in *A. protococcarum* were mapped with 25 pathways (Figure 5c). Interestingly, cluster II, featuring an upregulated trend over time, was enriched in glycosylphosphatidylinositol (GPI)-anchor biosynthesis, endocytosis, phagosome, ubiquitin-mediated proteolysis, SNARE interactions in vesicular transport, MAPK signaling pathway, and amino sugar and nucleotide sugar metabolism. 

### 2.9. Expression of CAZymes and Pathogen–Host Interaction Genes in A. protococcarum during Infection 

To assess the potential of *A. protococcarum* to depolymerize the cell walls of microalgal hosts, CAZymes-containing genes were searched using the *dbCAN2 meta server* (HMMER). There were 69 putative genes encoding CAZymes differentially expressed and identified in cluster II of *A. protococcarum*, which exhibited an upregulated temporal trend (see Table S3). CAZymes were classified into glycoside hydrolases (GHs), glycosyltransferases (GTs), and auxiliary activities (AAs) super families. Among them, two genes (Cluster-8366.7755 and Cluster-8366.85495) encoding α-glucosidase were upregulated by 6.2- and 10.7-log2 fold changes in LS, respectively. Two genes (Cluster-8366.11810 and Cluster-8366.64947) encoding trehalase and one gene (Cluster-8366.6141) encoding cellulase experienced a dramatic increase as well. The expression of genes encoding mannosyltransferase, galactosyltransferase, and glucosyltransferase was significantly upregulated during infection. It should be emphasized that several CAZymes genes associated with chitin binding and chitin synthase (AA15 and GT2) were also significantly upregulated, which might play a role in the chitin synthesis of A. protococcarum.

To predict key genes involved in infection, the pathogenicity genes of *A. protococcarum* were predicted using BLASTp against the Pathogen–Host Interaction database (PHI-base). In total, 1269 genes associated with key virulence and pathogenicity, accounting for 5.3% of the total predicted genes, were identified (see Table S4). Most genes (1231.97%) were expressed in the transcriptome of *A. protococcarum* during infection. Among them, 72 genes associated with increased virulence (Table S5) and 15 genes associated with effectors were significantly expressed at all infection stages (Table 2). Furthermore, 11 putative secretory protein-encoding genes were identified in pathogen–host interaction genes to seek for candidate virulence and effectors in *A. protococcarum*. However, only a candidate hypervirulence gene (Cluster-8366.11022) was upregulated by 2.1-, 5.9-, and 3.5-log2 fold change during infection. Two candidate effectors (Cluster-9881.0, Cluster-8366.6798) were upregulated by 2.4- and 2.5-log2 fold changes, during infection. 

## 3. Discussion

**Figure 6:**
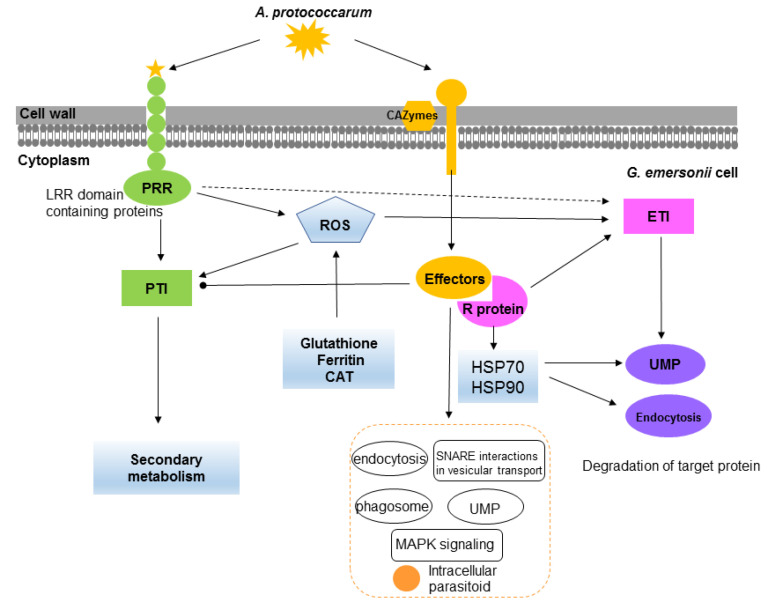
Schematic diagram showing the basic interaction of microalga *G. emersonii* and endoparasite *A. protococcarum*. Similar to plant, *G. emersonii* cells can perceive pathogen via potential PRRs and initiate PTI. Reactive oxygen species (ROS) was rapidly induced in response to infection. Meanwhile, ROS scavenging-related genes (glutathione, ferritin, CAT) were upregulated to eliminate excess ROS. Several HSP70 and HSP90 were upregulated and may serve as putative R genes to recognize the effectors of pathogens and trigger ETI. The ubiquitin–proteasome system (UPS) and endocytosis, which may be involved in regulating apoptotic cell death, were also upregulated to response to the infection. For endoparasite *A. protococcarum*, genes for carbohydrate-active enzymes, pathogen–host interaction, and putative effectors are significantly upregulated during infection. Endocytosis, phagosome, ubiquitin-mediated proteolysis, and SNARE interactions in vesicular transport pathway represent the KEGG enrichment pathways involved in the modulation of vacuole transport of intracellular parasitoid.

Mass cultivation of microalgae for biodiesel production is susceptible to epidemics caused by various pathogens [15]. Thus, it is essential to investigate microalgae–pathogen interactions. Dual RNA sequencing opens new prospects for investigating the underlying molecular mechanisms of microalgal host–pathogen interplay. However, dual transcriptional profiling of microalgae–pathogens is lacking. In this study, the experimental and stable pathosystem between oleaginous microalga *G. emersonii* and its endoparasite *A. protococcarum* was established. Using simultaneous dual transcriptomics, we dissected *G. emersonii* genes related to basic defense and *A. protococcarum* genes related to pathogenesis involved in the microalga–pathogen interaction. The basic interaction of host *G. emersonii* and endoparasite *A. protococcarum* is illustrated in Figure 6.

### 3.1. Defense Strategies in G. emersonii 

Plant defense against pathogenic fungi is based on a combination of the innate immune system, which includes a basal defense system based on pattern-recognition receptors (PRRs) that elicit PAMP-triggered immunity (PTI) and a more specialized recognition system based on resistance proteins (R proteins) that activate effector-triggered immunity (ETI) [16,17]. First, plant host immunity uses PRR, such as leucine-rich repeat kinases (LRR) and lysine motif (LysM) kinase receptors, to identify pathogens that attack the host. Transcriptome analysis of the red alga *P. yezoensis* during oomycete infection revealed that three lectin genes (PRRs) were upregulated after infection, whereas EGF- and LysM-containing genes could not be found [14]. The results show that microalga *G. emersonii* transcripts contained potential cellular receptors, including EGF, LRR, and LysM genes and that the expression of six LRR-containing genes was significantly upregulated in the infected *G. emersonii* cells. Second, plant resistance (R) proteins recognize the effectors of pathogens, activate signaling, and trigger ETI [18]. Recent studies have identified six genes encoding R proteins, including the nucleotide binding site (NBS) domain, that were identified in the genome of *Chromochloris zofingiensis*, and the fusion events of the NBS and LRR domains might occur in Chlorophyta and plants [19]. In *G. emersonii*, four genes encoding putative R proteins with an LRR domain showed no significant change during infection, indicating that endoparasite *A. protococcarum* can somehow avoid or inhibit this dense system. This might support the idea that ETI avoidance can be a crucial virulence strategy for plant pathogens [17]. Moreover, previous studies have confirmed the roles of large HSPs in recognizing plant pathogen effectors and activating defense responses against pathogens [13,14]. For instance, HSP70 and HSP90 might serve as R proteins that control plant disease resistance [20]. In our study, the genes encoding HSP70 and HSP90 were upregulated in *G. emersonii* during infection. Moreover, the genes encoding other key enzymes, such as HSP, which are involved in the endocytosis pathway, were induced. These results suggest that the upregulated genes encoding HSP70 and HSP90 might play a vital role in *G. emersonii* defense responses. 

The generation of ROS plays an important role in the PTI and ETI of plants as signaling molecules [21]. Recent study has demonstrated that PRR and co-receptors are required for ROS generation, which is a key signaling event combining PRR- and NLR-mediated immunity [22]. In this study, the ROS level gradually increased during infection, suggesting that *G. emersonii* has a signaling system that induces ROS in these cells when infected with fungal parasites. Based on STEM analysis, the genes in cluster VI were enriched with many oxidoreductase-related terms, which were upregulated or stable in the ES and MS stages and then downregulated in the LS stage. At the same time, plants might induce enzymes in response to excess ROS to protect their cells. It has been reported that microalgae can induce a variety of ROS scavenging enzymes due to oxidative stress [23]. Our data suggest that the genes associated with ROS-scavenging enzymes were induced in ES, MS, and LS. Notably, the expression of the genes encoding glutathione, ferritin, and CAT was upregulated dramatically, suggesting that the upregulation of ROS scavenging-related genes may be involved in defensive responses. These results denote that ROS production alone is insufficient to stop the attack of endoparasite *A. protococcarum,* as the ROS generation might also be affected by ROS scavenging-related genes. 

In plants, the ubiquitin–proteasome system (UPS) plays a crucial role in the control of plant immune signaling against pathogens [24]. In the UPS, damaged or superfluous proteins are mediated by three types of ubiquitin enzymes: E1 (ubiquitin-activating enzyme), E2 (ubiquitin-conjugating enzyme), and E3 (ubiquitin–protein ligase) [25]. Previous studies have concluded that ubiquitin-mediated proteolysis may be involved in regulating apoptotic cell death [26]. Our results show that cluster I was enriched in the ubiquitin-mediated proteolysis pathway. In *G. emersonii*, the expression of genes encoding enzymes E1 and E2 was significantly increased, especially in MS. In contrast, the genes related to E3 were found to be downregulated. These results hinted that UPS might be involved in the *G. emersonii* response to endoparasite *A. protococcarum*. Furthermore, recent findings have suggested that pathogens may manipulate the ubiquitin pathway of host cells to accelerate infection using effectors [27]. 

The expression of downstream TFs was also regulated. Several studies have reported that TF families, including WRKY, MYB, ERF, and bZIP, can play vital roles in signal transduction and plant defense [28,29]. In this study, SET, SNF2, GNAT, MYB, AP2-EREBP, and TRAF were the top six differentially expressed TFs in *G. emersonii*. However, only five TFs were upregulated during infection: two SNF2, one PLATZ, one FHA, and one orphan (Table 1). The TFs were differentially expressed in *G. emersonii* cells during infection, implying that these TFs might be required in the establishment of *G. emersonii* defense to endoparasite *A. protococcarum*. 

Together, the upregulation of these genes associated with response regulation suggests that *G. emersonii* has a conserved defense system against endoparasite *A. protococcarum*. 

### 3.2. Pathogenicity of A. protococcarum

For pathogenic fungi invading a host, carbohydrate-active enzymes (CAZymes) play important roles in penetration, colonization, exit, and dispersal during infection progress [30]. Several studies have indicated that massive CAZyme-encoding genes are activated during infection [31,32]. For endoparasite *A. protococcarum*, they need a living microalgal host to complete their life cycle (Ding et al., 2017). In other words, they need to use CAZymes to degrade the cell walls of *G. emersonii* to colonize. In this study, many CAZyme-encoding genes were differentially expressed and identified in cluster II of *A. protococcarum.* Notably, two genes encoding α-glucosidase were upregulated by 6.2- and 10.7-log2 fold changes in LS, respectively. Many genes encoding trehalase, cellulase, mannan, and several chitin-related genes also showed significant increases. Comprehensive GO enrichment and STEM results suggest that the genes in cluster III were enriched in the establishment of localization in cell and protein localization. In the LS, endoparasite *A. protococcarum* was widely distributed in the microalgal host, thus the expression of genes involved in the establishment of localization were decreased. This group of genes might play a positive role in the pathogenicity of *A. protococcarum*. 

Pathogens have unique virulence mechanisms that target and manipulate the host immune response to promote pathogenesis [33]. It is acknowledged that fungal pathogens can produce effectors to suppress PTI and ETI signaling and promote pathogen virulence [16], thus leading to successful proliferation. A recent study has suggested that pathogens can maximize pathogenicity benefits and avoid host detection through regulating effector dosage [17]. In *A. protococcarum*, 72 genes associated with increased virulence and 15 genes associated with effectors were upregulated in all the infection stages. Among them, 11 putative secretory protein-encoding genes were identified in the pathogen–host interaction genes. A candidate hypervirulence gene (Cluster-8366.11022) and two candidate effectors (Cluster-9881.0, Cluster-8366.6798) were upregulated during infection. These results suggest that these genes are involved in the pathogenicity of *A. protococcarum*. 

The comprehensive KEGG enrichment and STEM results suggest that the genes in cluster II, which showed an upregulated temporal trend, were closely related to endocytosis, phagosome, ubiquitin-mediated proteolysis, and SNARE interactions in vesicular transport pathways. These pathways may be involved in the modulation of vacuole transport, which is crucial for endoparasite survival and replication within the host [34]. Moreover, the mitogen-activated protein kinase (MAPK) signaling pathway was significantly enriched in cluster II. The MAPK signaling pathway is involved in the pathogenicity of plant pathogenic fungi [35]. Based on GO enrichment, genes related to the regulation of GTPase activity and response to host immunity, those genes that showed an upregulated temporal trend, were in cluster II. Given all the results together, we argue that endoparasite *A. protococcarum* possessed a robust pathogenicity, which can inhibit the expression of defense responses in *G. emersonii* to successfully infect the host. 

## 4. Materials and Methods

### 4.1. Microalga and Parasite Laboratory Culturing

The alga strain *G. emersonii* was acquired from the Algae Culture Collection at the Wuhan Botanical Garden, Chinese Academy of Sciences, China. *G. emersonii* cells were cultivated in modified BG11 medium, under laboratory conditions, at 26 ± 1 °C under 70 μmol photons m^−2^s^−1^ illumination for 14 h every day. 

The algal parasite *A. protococcarum* WZ01 was isolated from an open raceway pond of *G. emersonii* culture in Chenghai, China [11]. *A. protococcarum* WZ01 is an endoparasite and must be fed with *G. emersonii.* During the experiment, *A. protococcarum* WZ01 was maintained as a highly infective parasite through the addition of WZ01-infected *G. emersonii* filtrate (50 μL) to healthy *G. emersonii* culture (20 mL) every seven days. The dual-cultures were cultivated at 28 °C under illumination of 20 μmol photons m^−2^ s^−1^ with a 14 h:10 h light/dark cycle.

### 4.2. Infection Test and Sampling

*G. emersonii* cells were cultivated in 600 mL of modified BG11 medium in a photobioreactor with aeration of 1% (*v*/*v*) CO_2_ at 28 °C under illumination of 140 μmol photons m^−2^ s^−1^ with a 14 h:10 h light/dark cycle. Cells in the exponential growth phase were harvested and used for the infection experiments. The initial cell densities of *G. emersonii* in the tubular photobioreactors were OD_540_ = 0.10 ± 0.01. Then, 0.05% volume of the axenic *A. protococcarum* WZ01 filtrate through a 5 μm membrane filter (Whatman, Maidstone, UK) was added to the *G. emersonii* culture. Mixed cell cultures were cultivated under the same conditions and used as controls. The infected samples and controls had three replicates. 

After inoculation, the infection rate was determined using an Olympus BX51 microscope with hemocytometers. The infection rate was quantified as described by Ding et al. [4]. Samples were collected at 1, 2, 3, 4, and 5 days post-inoculation (dpi) for further analysis. 

### 4.3. Chlorophyll Fluorescence Parameter and ROS Measurement

During the experiments, the maximum Fv/Fm (maximum photochemical yield) was measured using a PAM 2500 fluorometer, as described in [36]. ROS levels in *G. emersonii* cells were detected using an ROS assay kit (S0033S, Beyotime, China). Briefly, 5 mL of mixed cell culture was centrifuged at 5000× *g* for 5 min, and the cell pellets were resuspended in 1 mL basal medium containing 10 μM DCFH-DA. The suspension was incubated in the dark at 37 °C for 30 min. After labeling, the cells were washed three times with basal medium and resuspended in the original culture medium. Subsequently, the cell suspension was transferred to each well of a black microtiter plate (Greiner Bio, Chimney well, Germany). Fluorescence was analyzed using a fluorescence microplate reader (TECAN, M200 PRO) with excitation and emission wavelengths of 488 and 525 nm, respectively. For each treatment and control, three replicates were performed.

### 4.4. RNA Isolation, cDNA Library Construction, and Sequencing

The control or infected samples (200 mL) were harvested by centrifuging at 5000 × g for 5 min. The cell pellets were frozen in liquid nitrogen and maintained at −80 °C until RNA extraction. Three replicates were prepared for each treatment and control. Total RNA from each sample was isolated using TRIzol reagent (Invitrogen, Carlsbad, CA, USA) according to the manufacturer’s instructions. Total RNA samples were quantified using a Nanodrop 2000 instrument (Thermo Scientific, San Jose, DE, USA) and the RNA Nano 6000 Assay Kit using the Agilent Bioanalyzer 2100 system (Agilent Technologies, Santa Clara, CA, USA) and Qubit RNA Assay Kit in Qubit 2.0 Fluorometer (Life Technologies, Carlsbad, CA, USA) and 1% agarose gels. A total of 1.5 μg RNA from each sample was used for cDNA library construction and RNA sequencing on an Illumina Novaseq 6000 platform with a 150 bp pair-ended strategy, which was performed at the Novogene Bioinformatics Institute (Beijing, China). 

### 4.5. Transcriptome Data Analysis

Raw reads were processed before downstream processing to derive clean reads by removing adaptor sequences, empty reads, and low-quality reads (< Q20). For the host, the clean reads were mapped to the *G. emersonii* reference genome using HISAT. For the pathogen, de novo transcriptome assembly was performed using Trinity [37] because of the lack of genomic information, and gene function annotation was performed in Nr, Nt, Pfam, KOG/COG, Swiss-Prot, KEGG, and GO databases. The host algae and endoparasite transcriptomes were subjected to differential expression analysis by calculating the target FPKM [38], thus allowing comparisons between different infection stages. Differentially expressed genes (DEGs) were identified using the DESeq method based on the Benjamini and Hochberg approach. The DEGs were deemed with a padj < 0.05 and ∣log_2_ (fold change)∣ >1 in sequence counts between the different comparison groups. Venn diagrams of host and fungal parasites were used to elucidate the differential gene expression patterns between different infection stages. 

Short Time-series Expression Miner (STEM) software was used for clustering analysis based on the similar expression trend patterns in *G. emersonii* and *A. protococcarum*. Novomagic, a free online platform, was used for GO enrichment, KEGG pathway analysis, and hierarchical clustering heatmap analysis (https://magic.novogene.com, Access date: 13 July 2021). The significantly enriched GO terms and KEGG pathways were determined with a *p*-value of < 0.05. To identify genes encoding CAZymes of *A. protococcarum* involved in the infection progress, we applied the dbCAN2 meta server (http://bcb.unl.edu/dbCAN2/blast.php, Access date: 22 July 2021) and Carbohydrate Active Enzymes database (http://www.cazy.org, Access date: 22 July 2021) with a BLAST e-value cutoff of 1 × 10^−5^ for analysis. Pathogen–host interaction genes were annotated using PHI-base [39]. PFAM annotation of each gene was performed using the Pfam database (http://pfam.xfam.org/, Access date: 23 July 2021). To predict secretory proteins, the SignalP 4.0 (Petersen et al. 2011), TMHMM v. 2.0, TargetP-2.0 Server, and kohgpi-1.5 programs (GPI-SOM database) were used. 

## 5. Conclusions

In summary, this is the first report on microalgae–endoparasite interaction at the transcriptional level. For microalgal hosts, STEM and functional analysis indicated that *A. protococcarum* infection triggers dynamic defense responses in the microalga *G. emersonii.* Potential pattern recognition receptors, ROS-scavenging enzymes, large HSPs, TFs, and the ubiquitin–proteasome system of *G. emersonii* all may be required for defense against endoparasite *A. protococcarum*. For endoparasite, transcriptome analysis revealed that the expression of genes associated with pathogenicity were upregulated significantly, such as CAZyme-encoding genes, PHI-base genes, and putative effectors. These results lay a foundation for understanding microalgal host defense strategies and parasite pathogenicity, which is crucial for preventing and controlling microalgal diseases.

## Figures and Tables

**Table 1:** Transcriptional factors of *G. emersonii* that were continuously upregulated because of infection.

No.	Gene ID	Types	Log2FC(ESvsGA)	Log2FC(MSvsGA)	Log2FC(LSvsGA)
1	gene-Cem00854	PLATZ	1.65	3.61	3.06
2	gene-Cem04263	SNF2	1.53	1.31	1.65
3	gene-Cem15036	FHA	2.91	2.45	1.58
4	gene-Cem16321	SNF2	2.08	3.57	2.51
5	gene-Cem19050	Orphans	1.41	2.68	3.18

Log _2_ FC = log _2_ fold change.

**Table 2:** Fifteen genes associated with effector in *A. protococcarum* were significantly upregulated in all the infection stages.

No.	Gene ID	ProteinID	PHI ID	Gene Name	Phenotype	log2FC(MS vs. ES)	log2FC(LS vs. ES)	log2FC(LS vs. MS)
1	Cluster-8366.85734	A0A0H3HVK0	PHI:5335	*clpV-5*	Effector	1.729	3.8033	1.8221
2	Cluster-8366.8318	Q8RP09	PHI:981	*hopI1*	Effector	2.0296	6.6465	4.3176
3	Cluster-8366.82670	Q79LY0	PHI:992/PHI:7237/PHI:7265	*hopPtoD2/hopAO1/HopPtoD2*	Effector	1.6504	4.9986	3.0935
4	Cluster-8366.80929	P17778	PHI:6101/PHI:6824/PHI:6830	*YopM*	Effector	6.0254	11.636	3.4623
5	Cluster-8366.7571	C5BD30	PHI:6294	*EseM*	Effector	2.5059	7.5318	4.6832
6	Cluster-8366.72579	Q8PC98	PHI:7945	*pip*	Effector	4.4021	9.7346	2.9476
7	Cluster-8366.6633	Q8PC98	PHI:7945	*pip*	Effector	3.5808	10.373	4.6194
8	Cluster-8366.43382	Q8XTK9	PHI:5119	*RSp0099*	Effector	1.7086	4.8665	2.9109
9	Cluster-8366.14241	P17778	PHI:6101/PHI:6824/PHI:6830	*YopM*	Effector	1.6503	5.6023	3.6416
10	Cluster-8366.11799	Q8PI08	PHI:2703	*xac3090*	Effector	1.5342	5.0159	3.2262
11	Cluster-8366.11711	Q8XZN9	PHI:5173	*RSc1356*	Effector	2.971	6.0476	2.8247
12	Cluster-8366.11710	Q8XZN9	PHI:5173	*RSc1356*	Effector	2.3996	5.6692	3.0096
13	Cluster-8366.11150	Q8XZN9	PHI:5173	*RSc1356*	Effector	3.3433	7.5956	3.9299
14	Cluster-8366.11149	Q8XZN9	PHI:5173	*RSc1356*	Effector	3.8592	11.787	3.2499
15	Cluster-8366.11148	Q8XZN9	PHI:5173	*RSc1356*	Effector	3.1417	7.4909	4.0076

## Data Availability

The RNA sequencing read data were deposited in the GenBank SRA database under the accession number PRJNA753980.

## Supplementary Materials

The following are available online at https://www.mdpi.com/article/10.3390/ijms222312847/s1.

## List of Abbreviations

CAZymes	carbohydrate-active enzymes
DEGs	differentially expressed genes
EGF	epidermal growth factor
ETI	effector-triggered immunity
ES	early stage
Fv/Fm	maximum photochemical yield
GA	control
HSPs	large heat shock proteins
LRR	leucine-rich repeat
LysM	lysine motif
LS	late stage
MS	medium stage
MAPK	mitogen-activated protein kinase
NBS	nucleotide binding site
PTI	PAMP-triggered immunity
PRRs	pattern recognition receptors
R proteins	resistance proteins
ROS	reactive oxygen species
STEM	short time-series expression miner
UPS	ubiquitin–proteasome system
