# Supplementary material for: Physiological and Dual Transcriptional Analysis of Microalga Graesiella emersonii–Amoeboaphelidium protococcarum Pathosystem Uncovers Conserved Defense Response and Robust Pathogenicity
Source: Int J Mol Sci. 2021 Nov 27;22(23):12847. doi: 10.3390/ijms222312847 (PMC8657485; doi:10.3390/ijms222312847)
Supplement: Supplementary file 1 [file ijms-22-12847-s001.zip › ijms-1460499-supplementary/Supplementary Materials/Supplementary materials.pdf]

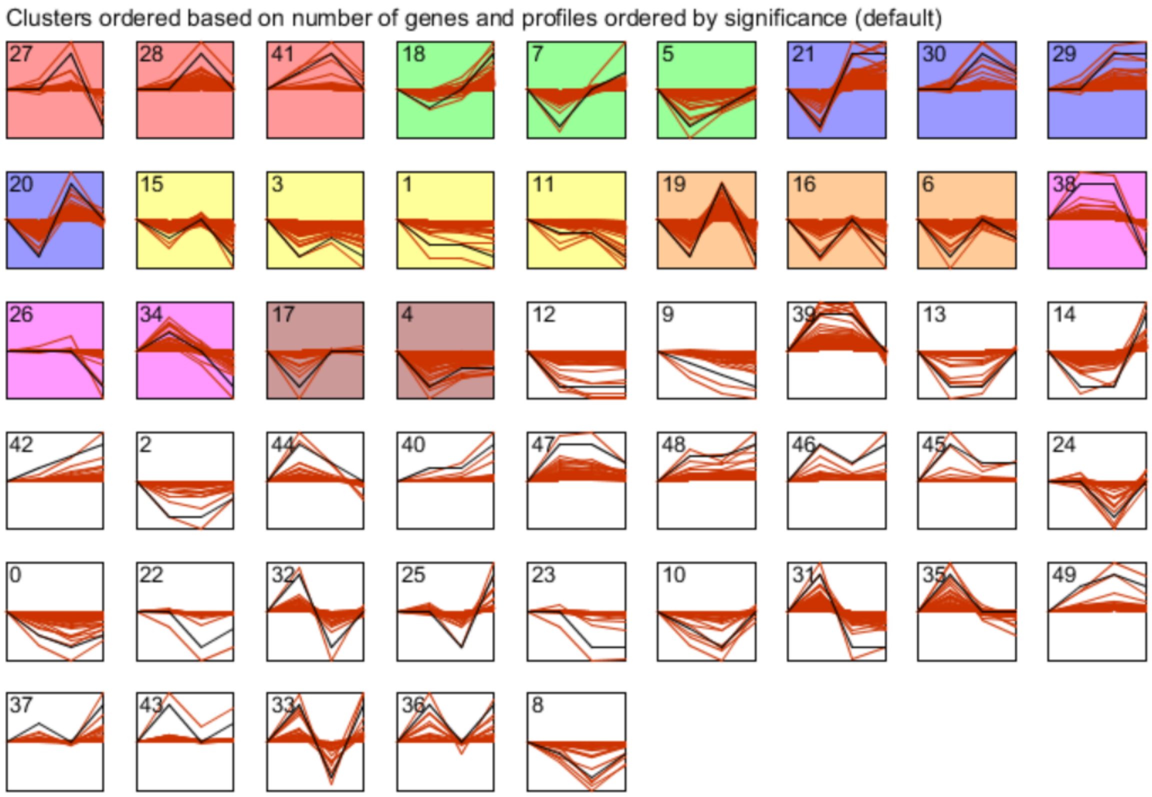

Figure S1. a total of 50 profiles in G. emersonii were generated by STEM analysis.

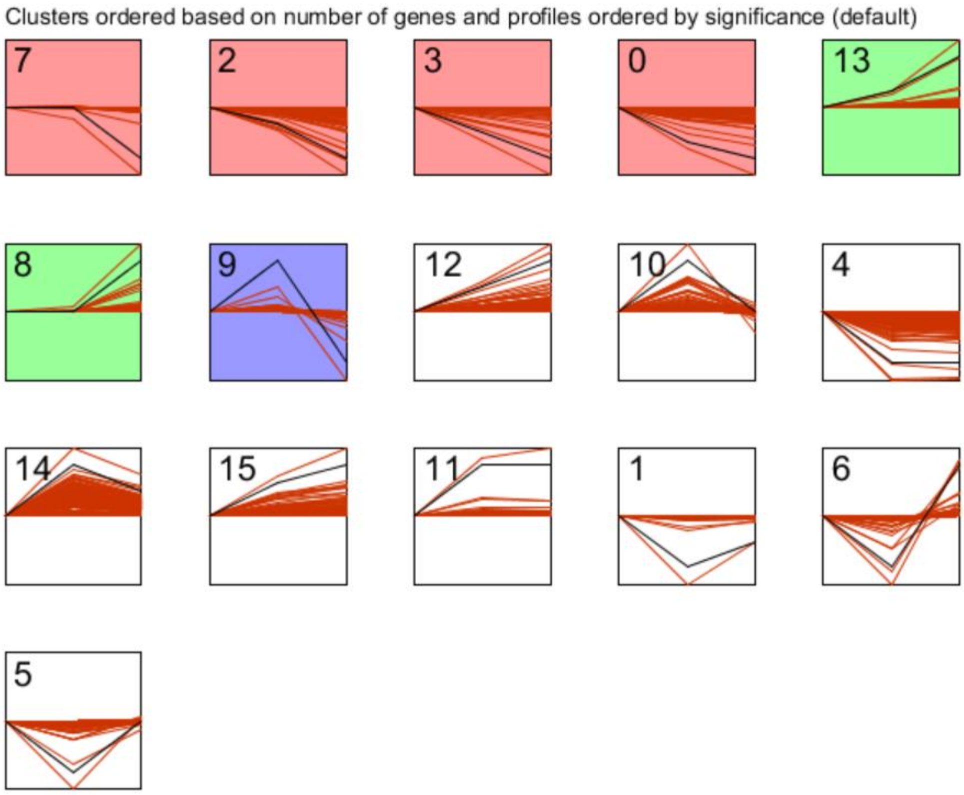

Figure S2. a total of 16 profiles in *A. protococcarum* were generated by STEM analysis.

Table S1. summary of sequencing data quality.

Sample Name	Raw Reads	Clean Reads	Clean Bases	Error Rate (%)	Q20 (%)	Q30 (%)	GC Content (%)
GA1	56713438	56586746	8.49G	0.02	98.49	95.58	52.98
GA2	57412792	57268784	8.59G	0.02	98.43	95.42	53.01
GA3	53084304	52959550	7.94G	0.02	98.45	95.48	52.98
ES1	63678132	63527840	9.53G	0.02	98.50	95.56	53.12
ES2	58709598	58565036	8.78G	0.02	98.52	95.64	53.17
ES3	51318582	51219086	7.68G	0.02	98.64	95.90	53.31
MS1	51582250	51476854	7.72G	0.02	98.53	95.62	51.20
MS2	55264588	55144438	8.27G	0.02	98.46	95.47	51.52
MS3	58004912	57881082	8.68G	0.02	98.43	95.37	51.45
LS1	52364196	52266404	7.84G	0.02	98.40	95.18	46.99
LS2	58822838	58697036	8.8G	0.02	98.36	95.16	47.21
LS3	67691368	67542428	10.13G	0.02	98.41	95.31	46.90
